# 3D printing and virtual planning in anterior mandibular fixation: A clinical study

**DOI:** 10.6026/973206300212116

**Published:** 2025-07-31

**Authors:** Kishankumar Avula, Taskeen Kaur Sethi, Saumil Bavaliya, Shreya Jawalkar, Piyush Sharma, Mazen Ahmad Almasri, Heena Dixit

**Affiliations:** 1 Department of Oral and Maxillofacial surgery, Anil Neerukonda Institue of Dental Sciences, Visakhapatnam andhra Pradesh, India; 2 Department of Oral and Maxillofacial Surgery, Gian Sagar Dental College and Hospital, Punjab, India; 3 Dental Surgeon, Government Dental College Jamnagar, Ahmedabad, Gujarat, India; 4 Department of Oral And Maxillofacial Surgery, C.K.S teja Dental College and research institute, Tirupati andhra Pradesh, India; 5 Consultant Maxillofacial and Facial Plastic Surgeon, Xiyan Plastic Surgery Hospital Shanghai, China; 6 Department of Oral Maxillofacial Surgery, King Abdulaziz University, Saudi Arabia; 7 Department of Clinical Psychology, Andhra University, Visakhapatnam, Andhra Pradesh, India

**Keywords:** 3D printing, mandibular fracture, virtual surgical planning, maxillofacial trauma, plate fixation

## Abstract

Anterior mandibular fractures present a unique challenge due to complex curvature and aesthetic demands. Thirty patients with isolated anterior mandibular fractures were prospectively divided into two groups. Group B demonstrated significantly reduced operative time (68.3 ± 8.9 minutes vs. 92.6 ± 10.4 minutes, p < 0.001) and fewer intraoperative adjustments. Complication rates were lower in Group B, though not statistically significant. Thus, 3D virtual planning and printing enhance surgical accuracy and reduce operative time in anterior mandibular fixation.

## Background:

Mandibular fractures, particularly those involving the anterior region, represent a significant portion of maxillofacial trauma cases and often result from high-impact injuries such as road traffic accidents, physical assaults, or falls. Fixation of these fractures is critical to restore functional occlusion, aesthetics and mandibular continuity. Conventionally, reduction and fixation of mandibular fractures have relied on intraoperative judgment and two-dimensional radiographic planning, both of which are associated with challenges in accuracy, intraoperative time and risk of complications [1, 2- 3]. In recent years, the integration of digital technologies such as 3D virtual surgical planning (VSP) and 3D printing has shown promising advancements in the field of craniofacial trauma surgery. These tools allow for precise preoperative visualization, simulation of osteosynthesis and fabrication of patient-specific anatomical models and surgical guides. Their use has enhanced preoperative communication, reduced guesswork during surgery and facilitated a more accurate transfer of the surgical plan to the operating room [4, 5- 6].

In anterior mandibular fractures, where the curvature and limited bone stock add to the complexity, accurate adaptation of fixation plates is paramount for optimal healing outcomes. Despite the increasing use of these technologies, limited clinical data exist comparing the intraoperative and postoperative accuracy of conventional fixation methods with 3D-assisted approaches in the anterior mandible. Studies have suggested potential reductions in operative time, improved plate adaptation and decreased complication rates with the aid of 3D planning and printed models [7, 8- 9]. Specific focus is placed on the precision of plate placement, operative time and early postoperative complication rates [10]. However, systematic evaluation through clinical trials remains essential to validate these claims and to quantify the magnitude of improvement, if any, in surgical outcomes. Therefore, it is of interest to evaluate the accuracy of 3D printing and virtual planning in the fixation of anterior mandibular fractures by comparing outcomes with conventional methods.

## Materials and Methods:

This prospective clinical research was conducted over a period of 18 months in the Department of Oral and Maxillofacial Surgery at a tertiary care center. Ethical clearance was obtained from the institutional ethics committee and informed consent was collected from all participants. A total of 30 patients diagnosed with isolated anterior mandibular fractures were enrolled and randomly divided into two groups: Group A (n=15) underwent conventional open reduction and internal fixation (ORIF), while Group B (n=15) underwent fixation using preoperative virtual surgical planning and 3D-printed anatomical models.

## Inclusion criteria:

Patients aged 18-60 years with anterior mandibular fractures involving the symphysis or parasymphysis regions and without commination or infection.

## Exclusion criteria:

Patients with systemic conditions affecting bone healing, edentulous mandibles, or associated midface fractures. In Group A, standard ORIF was performed using titanium miniplates based on surgeon experience and intraoperative adaptation. In Group B, patients underwent preoperative "cone-beam computed tomography (CBCT)" scanning. The DICOM data were used for virtual planning using proprietary planning software. 3D-printed models were created using "fused deposition modeling (FDM)" technology. Titanium plates were pre-contoured on the models before surgery and transferred intra-operatively. Parameters evaluated included operative time, accuracy of plate adaptation (measured by postoperative CT superimposition analysis), intraoperative adjustments and early complications such as infection, malocclusion, or hardware exposure. All patients were followed up for a minimum of 3 months. Statistical analysis was performed using SPSS v26. Independent t-tests and chi-square tests were applied with significance set at p < 0.05.

## Results:

A total of 30 patients (20 males and 10 females) were included in the research with a mean age of 34.2 ± 8.7 years. Group A (conventional fixation) and Group B (3D-assisted fixation) were comparable in baseline characteristics, including fracture type and patient demographics. Mean operative time in Group B (3D-planned group) was significantly shorter compared to Group A. Group B also required fewer intraoperative adjustments to plate contouring and positioning. Specifically, Group A required intraoperative plate bending in 12 out of 15 cases (80%), whereas only 3 out of 15 cases (20%) in Group B needed such adjustments (p < 0.01) (Table 1 - see PDF). Postoperative CT scans were evaluated for accuracy of plate adaptation by superimposing preoperative plans with postoperative images. Deviation from planned plate position was significantly lower in Group B. The incidence of early complications such as infection, malocclusion and hardware exposure was lower in the 3D-assisted group but not statistically significant (Table 2 - see PDF). These results indicate that virtual surgical planning combined with 3D-printed modeling enhances surgical accuracy and efficiency in anterior mandibular fracture fixation.

## Discussion:

The present clinical research demonstrates that the application of virtual surgical planning and 3D printing in anterior mandibular fixation significantly enhances the precision of surgical outcomes compared to conventional techniques. Notably, the reduction in operative time and intraoperative plate adjustments observed in the 3D-assisted group highlights the practical utility of these technologies in streamlining complex procedures. Virtual planning allows surgeons to simulate osteosynthesis in a digital environment prior to the actual operation, thereby minimizing intraoperative uncertainty. The current research showed a statistically significant reduction in operative time (approximately 24 minutes on average), aligning with previous findings that have linked digital planning with decreased surgical duration and more efficient operating room use [11]. The precision of pre-contoured plates based on 3D-printed models ensured better adaptation to mandibular anatomy, which in turn minimized the need for intraoperative modifications-a factor that can reduce soft tissue trauma and overall surgical fatigue. Accuracy of plate placement, measured by the deviation from the planned trajectory, was significantly better in the 3D group. Such accuracy is critical in the anterior mandible due to its aesthetic and functional importance. Even small deviations in plate positioning can impact occlusion, facial symmetry and bone healing [12]. Moreover, enhanced accuracy may lead to reduced postoperative complications, although in the present research, the lower complication rates in the 3D group were not statistically significant due to limited sample size. While the upfront cost of 3D printing technology may be considered a limitation, studies have shown that the long-term benefits-including reduced operative time, fewer revisions and lower complication rates-can potentially offset these initial investments [13]. Furthermore, as additive manufacturing technology becomes more accessible and cost-effective, its integration into routine clinical workflows is expected to increase, especially in centers dealing with high volumes of craniofacial trauma [14]. It is also important to consider the learning curve associated with digital workflows. Surgeons need training in software use and interpretation of virtual simulations to ensure that the benefits of technology translate effectively to clinical outcomes. Nevertheless, the evidence supports that 3D-assisted mandibular fixation can significantly improve surgical accuracy and efficiency [15, 16, 17, 18, 19-20].

## Conclusion:

3D virtual planning and 3D printing significantly improve the accuracy and efficiency of anterior mandibular fracture fixation. Incorporating these digital tools into routine practice can enhance surgical outcomes, particularly in anatomically complex or aesthetically sensitive regions like the anterior mandible.
